# To aggregate or not to aggregate high-dimensional classifiers

**DOI:** 10.1186/1471-2105-12-153

**Published:** 2011-05-13

**Authors:** Cheng-Jian Xu, Huub CJ Hoefsloot, Age K Smilde

**Affiliations:** 1 Biosystems Data Analysis group, University of Amsterdam, P.O. Box 94215 1090 GE Amsterdam, The Netherlands

## Abstract

**Background:**

High-throughput functional genomics technologies generate large amount of data with hundreds or thousands of measurements per sample. The number of sample is usually much smaller in the order of ten or hundred. This poses statistical challenges and calls for appropriate solutions for the analysis of this kind of data.

**Results:**

Principal component discriminant analysis (PCDA), an adaptation of classical linear discriminant analysis (LDA) for high-dimensional data, has been selected as an example of a base learner. The multiple versions of PCDA models from repeated double cross-validation were aggregated, and the final classification was performed by majority voting. The performance of this approach was evaluated by simulation, genomics, proteomics and metabolomics data sets.

**Conclusions:**

The aggregating PCDA learner can improve the prediction performance, provide more stable result, and help to know the variability of the models. The disadvantage and limitations of aggregating were also discussed.

## Background

The mining of high-dimensional data in which the number of features is much larger than the number of samples, has become increasingly important, especially in genomics, proteomics, biomedical imaging and other areas of systems biology [1]. The availability of high dimensional data along with new scientific problems have significantly challenged traditional statistical theory and reshaped statistical thinking [2].

The high dimensionality of functional genomic data sets poses problems to build classifiers. Because of the sparsity of data in high dimensional spaces, many classical methods of classification break down. For example, Fisher discrimination rule will be inapplicable because the within scatter matrix become singular if the number of variables is larger than the number of samples [3,4].

Another problem is caused by the small sample size. The number of samples is usually not adequate to be representative of the total population. Moreover classifiers built on small sample sets are often not stable and may have a large variance in the number of misclassification [5]. One common approach for this problem is to aggregate many classifiers instead of using a single one. There has been considerable interest recently in the application of aggregating methods in the classification of high-dimension data [6-11]. The most well-known method in this class of techniques is perhaps bootstrap aggregating (bagging). Breiman found that gains in accuracy could be obtained by bagging when the base learner is not stable [6]. However, Vu and Braga-Neto argued that the use of bagging in classification of small-sample data increases computational cost, but is not likely to improve overall classification accuracy over other simpler classification rules [10]. Moreover, if the sample size is small, the gains achieved via a bagged ensemble may not compensate for the decrease in accuracy of individual models [11].

Cross-validation is probably the most widely used method for estimating prediction error. In small sampled high dimension data modeling, *k*-fold cross-validation is often used [1]. The *k*-fold cross-validation estimate is a stochastic variable that depends on the partition of the data set. Full cross-validation, that means performing all-possible ways of partitioning, will give an accurate estimation, but is computationally too expensive. Therefore, repeating *k*-fold cross-validation multiple times using different splits provides a good Monte-Carlo estimate of the full cross-validation [12]. This repeating procedure results in a lot of classifiers.

In this paper, we aggregated the classifiers obtained from principal component discriminant analysis (PCDA) with a double cross-validation scheme [13]. PCDA is an adaptation of Fisher's linear discriminant analysis (FLDA) for high-dimensional data. In PCDA, the dimensionality of the data is reduced by principal component analysis (PCA). In the reduced dimensional space the within scatter matrices is nonsingular and classical LDA can be performed [13-16]. A double cross-validation scheme was used to estimate both the number of principal components and the predictor error of the PCDA model [17]. The classifiers that were obtained from the different cross-validation loops are aggregated to make a single classifier. This approach is tested on simulated data, gene expression, proteomics and metabolomics data. The results obtained from the research may provide insights into the use of aggregating learner in low sample, high dimensional biological data.

## Methods

### PCDA

Given a high dimensional data set **A **of size *m *× *n*, where *m *is the number of samples and *n *is the number of features (*m << n*), classical FLDA [18] finds the discriminating direction **d**_*n* × 1 _that maximizes the ratio of the between-class scatter **S**_*b *_against the within-class scatter **S**_*w*._(1)(2)(3)

Here *r *is the number of classes, and each class has *m*_*i *_samples. *M*_*i *_is the index set of samples in each class *i*.  and  are the class centroids and the global centroid respectively.

The discriminating direction **d **is the eigenvector corresponding to the largest eigenvalue of the matrix . Because the number of features *n *is larger than the number of samples *m *in high dimensional data, the matrix **S**_***w ***_is singular. This means that  does not exist and FLDA cannot be applied directly.

To overcome the difficulties imposed by the singular covariance matrices, the data can be first projected onto a low dimension PCA subspace, and LDA is then applicable in this PCA subspace. The main goal of PCA is to reduce the dimensionality of a data, whilst retaining as much as possible of the information present in the original data. This reduction is achieved by a linear transformation to a new set of variables, the principal component (PC) scores. The combination of LDA with PCA yields principal component discriminant analysis (PCDA).

### Aggregating PCDA with double cross-validation

**Figure 1:**
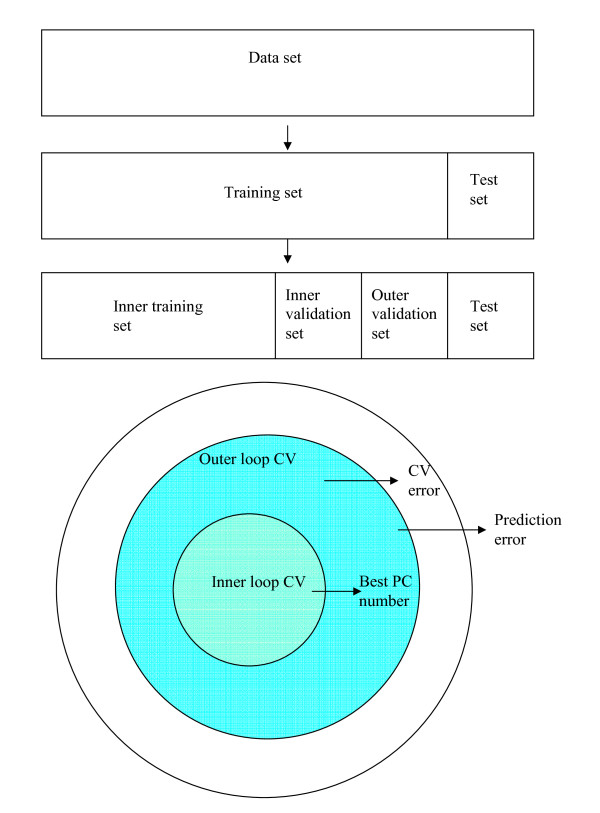
**The partition of a data set for model selection and the estimation of the cross-validation error and prediction error**. In the inner loop cross-validation, inner training set and inner validation set are used to determine the number of principal components (PC), and the model is fit on the inner training set. In the outer loop cross-validation, the model is built on the inner training set and the inner validation set, and an outer validation set are used to estimate the cross- validation error. In prediction, the model is built on the inner training set and the inner validation set, and the test set is used to obtain the prediction error.

The optimal number value of reduced dimensions of PCA is usually determined by cross-validation. The simplest form of cross-validation is to split the data randomly into *K *mutually exclusive parts, building a model on all but one part, and to evaluate the model on the omitted part. This strategy allows for estimating the optimal model complexity; however, the resulting prediction performance estimate is often too optimistic since the same samples were also used to find the best number of PC's and thus they are not completely independent. It is therefore recommended to use a double cross-validation approach [13,17,19,20]. As shown in Figure 1, first the original data set was divided into two parts, training set and test sets. The test set was not used in double cross-validation scheme and it was employed afterwards to evaluate how good the built classifier really is. The training set was partitioned into *K *parts. Of the *K *parts, a single part is retained as the outer validation set, and the remaining *K*-1 subsamples are used as inner training data and inner validation set. On the remaining *K*-1 parts, a *K*-1-fold cross-validation is performed to find the best number of PC components. This is a nested validation scheme. The inner validation set is used to determine the optimal number of principal components, and the outer validation set is used to find the cross-validation error of the method. In summary, the double cross-validation with PCDA is summarized in the following pseudo code

Divide the training data set into *K *parts:

For *i*= 1 to *K*

   For *j*= 1 to *K*-1

      Build PCDA models with different PCs

   End

   Find an optimal PC number

   Build PCDA model with the optimal PC number

End

   Obtain cross-validation error.

Since the cross-validation error accuracy would depend on the random assignment samples, a common practice is to stratify the folds themselves [12]. In stratified *K*-fold cross-validation, the folds are created in a way that they contain approximately the same proportion of classes as the original dataset. With randomly chosen partitions of inner and outer validation set, we can repeat the double cross-validation scheme to produce a lot of PCDA classifiers. The multiple versions of the predictors can be aggregated by majority voting, i.e., the winning class is the one being predicted by the largest number of predictors.

## Data

### Simulation

The simulated data contain two classes. Each class L_*i *_(*i*= 1, 2) consists of 100 objects and each object has 590 features, and it is sampled from a multivariate normal distribution *N *(**v**_*i*_, **Ω**) respectively, *i *= 1, 2. Here **v**_*i *_is the mean of class L_*i*_, and **Ω **represents the covariance of the simulated data. To make the simulation more closely to real data, we constructed the simulated data from the Gaucher proteomics data (see below). Suppose the means and covariances of two classes in the auto-scaled Gaucher data are represented by vector **u**_1_, **u**_2_, matrix **Ω**_1_, and **Ω**_2 _respectively, and the mean **v**_1 _and **v**_2 _and covariance matrix **Ω **of the simulated data were calculated by the following equations.(4)(5)(6)

Equation 4 and 5 are to ensure the separability of two classes, and equation 6 is to make two classes have the same common covariance matrix **Ω**.

By following the above procedure, we obtain the simulated data set of size 200 × 590. Before building PCDA classification model by double cross-validation on the simulated data set, we separated the simulated data set into training set and test set as shown in Figure 1. In order to form training sets of differ sample sizes, we randomly selected 12, 30, 50, 75, 100 objects from 200 objects. In the test set, 100 objects were random selected without replacement from the data set after removing the training set. The whole selection procedure was repeated 100 times randomly. To make a reasonable comparison, we fix the random seeds in each selection procedure. In single PCDA, a double cross-validation with ten-fold in the outer loop and nine-fold in the inner loop were used to obtain the optimal PC number and cross-validation error. In aggregating PCDA, the PCDA approach was repeated 51 times with different cross-validation splits to obtain an aggregated classifier. Besides, we also constructed a single PCDA model with double cross-validation in the simulated data sets to compare the classification performance of PCDA with aggregating PCDA.

### Leukemia gene expression data

Leukemia data from high-density Affymetrix oligonucleotide arrays were previously analyzed in Golub and Tibshirani [21,22], and are available at http://www.broad.mit.edu/cgi-bin/cancer/datasets.cgi. There are 7129 genes and 72 samples coming from two classes: 47 in class ALL (acute lymphocytic leukemia) and 25 in class AML (acute mylogenous leukemia). Among these 72 samples, 38 (27 in class ALL and 11 in class AML) are set to be training samples and 34 (20 in class ALL and 14 in class AML) are set as test samples. The data is mean-centered before classification. It should be noted that the pretreatment step such as mean-centering and auto-scaling was always performed on the training data and then the test data was pretreated with by the mean and standard deviation obtained from the training set. Auto-scaling means mean-centering the data and scaling each column by its standard deviation.

### Gaucher proteomics data

The data consist of serum protein profiles of 20 Gaucher patients and 20 controls [13]. Serum samples were surveyed for basic proteins with SELDI-TOF-MSS making use of the anionic surface of CM10 PrtoeinChip. All preprocessing (spot-spot calibration, baseline subtraction, peak detection) of the SELDI-TOF-MS data was performed using Ciphergen software. The data set of size 40 × 590 is available at http://www.bdagroup.nl/content/Downloads/datasets/datasets.php. One Gaucher sample (a female receiving enzyme replacement therapy) has been detected as an outlier and was removed. The spectra profiles were first normalized by dividing each profile by its median to arrive at comparable spectra. Subsequently, the data sets were auto-scaled before classification.

### Grape extract metabolomics data

The data set is from Unilever Food and Health Research, Vlaardingen, Netherlands, Thirty five healthy males were recruited to investigate the effect of grape extract supplementation on vascular function and other vascular health markers. The study has a double-blind, placebo controlled randomized full crossover design with 3 treatments, a run-in period, 3 interventions- and 2 washout periods. 1D 1H NMR spectra of plasma: D2O (1:1 v/v) samples were recorded on a Bruker Advance 600 MHz NMR spectrometer according to a Standard Operating Procedure with a pulse sequence. All data were processed in Bruker XWIN-NMR software version 3.0 (Bruker BioSpin GmbH, Rheinstetten, Germany) and imported in AMIX software from Bruker. Due to some missing data, the final NMR data of 276 plasma samples were bucketed in the spectral region 0-9 ppm using a bucket-width of 0.02 ppm.

The data set of size 276 × 412 of two classes was divided into two subsets, 200 samples in training set and 76 samples in prediction set, using the Kennard-Stone method [23]. The Kennard-Stone method was used to select objects to model such that they are uniformly scattered over the experimental space. In the training set and test set, the samples were assigned in such a way that the ratio of class membership is similar to the original data. The data sets were auto-scaled before classification.

## Results and Discussion

### When aggregating works

**Figure 2:**
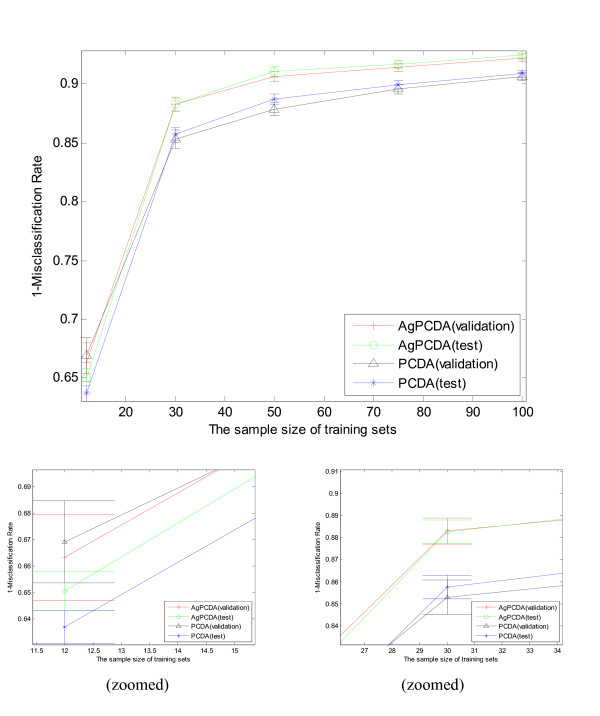
**Learning curves of aggregating PCDA and PCDA**. With the increasing of sample size for 12 to 100, the classification performance of aggregating PCDA and PCDA is increased significantly, and the variation of classification models also tends to be reduced. The classification performance is represented by 1 minus misclassification rate, and the variation of classification performances is represented by an error bar. The upper error ranges for each point in error bar is obtained with adding standard deviation of mean of classification performance and lower error ranges is obtained with subtracting standard deviation of mean of that. The figure shows that aggregating PCDA usually gives a better classification performance than PCDA. The classification of validation sets and test sets are quite similar since two sets follow the same distribution.

Breiman [6] has noticed that the efficiency of aggregating depends on the stability of the prediction or classification rule. Each cross-validation PCDA classifiers are constructed on different samples, so it is expected that there will be some variance in the prediction error. First, we applied aggregating PCDA on the simulated data sets. As shown in Figure 2, the classification performance of aggregated PCDA is usually better than that of PCDA itself. Here, the single PCDA itself uses ten fold outer cross-validation to determine the cross-validation errors and a nine fold inner cross-validation to determine the optimal number of principal components. The aggregated PCDA was constructed by repeating single PCDA 51 times. The simulation results in Figure 2 themselves are a pro of aggregating. As the training sets and prediction sets follow same distribution, the cross- validation error and prediction error are quite similar in Figure 2. A close look on Figure 2 also tells us, when the number of sample size is increasing, the classification rate is increased and the variation of the prediction error is reduced.

**Figure 3:**
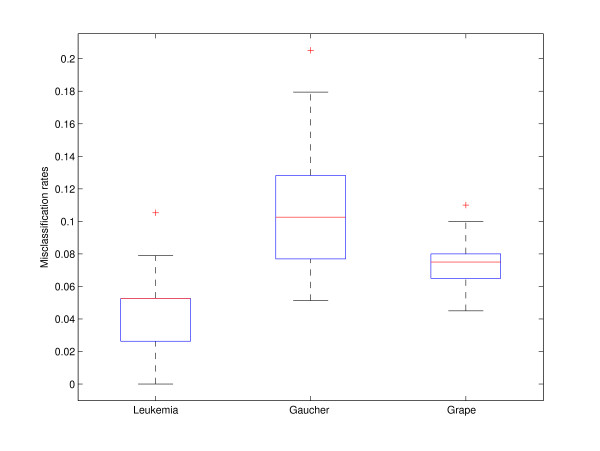
**Boxplot of cross-validation errors for three real data sets**. Misclassification rates are obtained from 1000 times repeating 10 fold double cross-validations. The ratios of feature to samples in training sets are 38/7129 (Leukemia), 39/500(Gaucher) and 200/412 (Grape). The most stable case is from grape data, and the ratio of feature to sample is the lowest among all three data sets.

**Figure 4:**
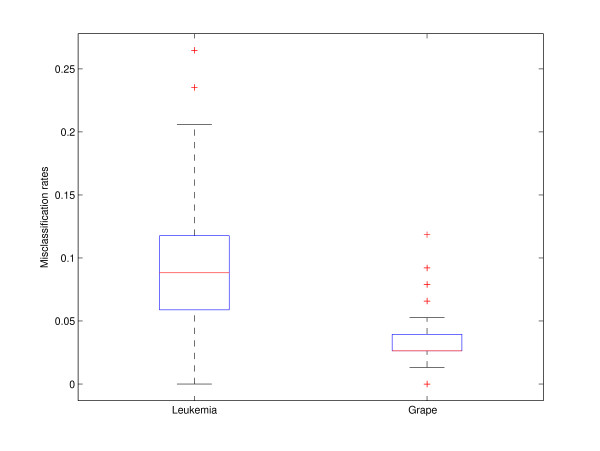
**Boxplot of prediction errors for two real data sets**. Misclassification rates are obtained from 1000 times repeating 10 fold double cross-validations. The ratios of features to samples in test sets are 34/7129 (Leukemia) and 76/412 (Grape). The model of grape data is more stable since the ratio of feature to sample is lower.

We further applied PCDA and aggregated PCDA on three real data sets. Figures 3 and 4 illustrate the variation of misclassification rate of the data sets in training and predictions.

The aggregated PCDA was constructed by repeating PCDA 1000 times. As shown in Figures 3 and 4, the median of the misclassification rate is indicated by the center line, and the first and third quartiles are the edges of the box area, which is known as the inter-quartile range. The extreme values (within 1.5 times the inter-quartile range from the upper or lower quartile) are the ends of the lines extending from the inter-quartile range. Points at a larger distance from the median than 1.5 times the inter-quartile range are plotted individually as plus sign. Due to the low sample size in the Gaucher data, a separate test set was not created. There are only two data sets giving the performance of the prediction of the test set in Figure 4. Obviously, the variations in the error rate of the PCDA models are quite large in the data sets, especially when ratio of feature to sample is high. The most stable case is from the grape data, and the ratio of feature to sample is the lowest among all three data sets. Table 1 and Table 2 also show that aggregating PCDA model often gives an improved performance over a single PCDA model in the three real data sets. In Table 1 and Table 2, the performance of a single PCDA is represented by the median of the misclassification rate.

**Table 1:** Cross-validation errors evaluated by outer validation sets with PCDA

Misclassification rate	PCDA	Aggregating PCDA
Leukemia	2/38	1/38
Gaucher	4/39	4/39
Grape	15/200	12/200

**Table 2:** Prediction errors evaluated by test sets with PCDA

Misclassification rate	PCDA	Aggregating PCDA
Leukemia	3/34	0/34
Grape	2/76	2/76

The aggregated PCDA can make a good PCDA classifier better since the variance of misclassification rate can be reduced [24-27]. A heuristic explanation is that the variance of the prediction error of the aggregated classifier is equal to or smaller than the error of the original classifier since majority voting is modeling averaging.

The dimension reduction step by PCA can not be guarantied to preserve all directions that contain discriminative information [28]. But in an aggregated PCDA model, the discarded discriminant information of one PCDA model can be re-modeled from other PCDA model with different partition of training data sets by cross-validation. So, aggregating PCDA itself may contain more discrimination information than single PCDA.

We also compared PCDA with the Support Vector Machine (SVM) classifier [29], and the results are shown in Table 1, 2, 3, 4. We found that the single PCDA classifier has a comparable result to the single SVM classifier. However, aggregating PCDA achieves better results than SVM, PCDA, and aggregating SVM classifiers.

**Table 3:** Cross-validation errors evaluated by outer validation sets with SVM

Misclassification rate	SVM	Aggregating SVM
Leukemia	2/38	2/38
Gaucher	4/39	4/39
Grape	16/200	15/200

**Table 4:** Prediction errors evaluated by test sets with SVM

Misclassification rate	SVM	Aggregating SVM
Leukemia	3/34	3/34
Grape	3/76	3/76

### When aggregating does not work

Aggregating may increase the bias of a learner since only a part of the training data are sampled by cross-validation or bootstrapped for modeling. That is to say, the use of *K*-fold cross-validation may have a negative effect on the accuracy of individual PCDA models. As shown in Figure 2, when the sample size is twelve, the performance of PCDA classifier is relatively bad and not stable. After aggregating, the classification performance did not achieve expected training and prediction performance yet, since basically in such case more samples are needed to build a precise model. Another situation which does not favor aggregating is case of very weak learners. A very weak learner means that the performance of learner is even worse than random guess. Aggregating such learner will make prediction even worse because averaging such learners will result in a learner that will give a wrong prediction in all cases. For example, if an observation is classified as a success about four times out of ten. After the majority voting, it will give 100% wrong.

### Further notes

**Figure 5:**
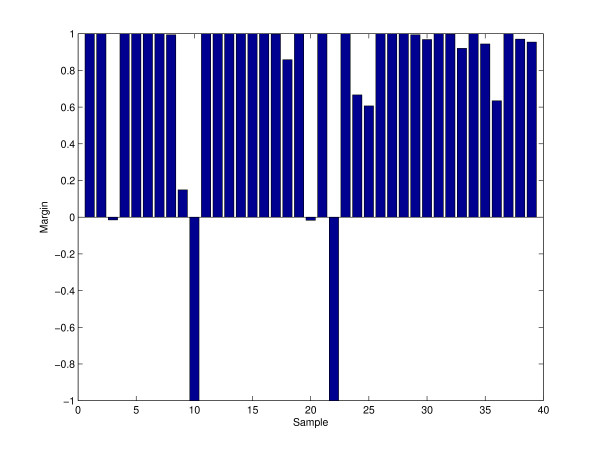
**Margins plot of thirty-nine samples in Gaucher data**. The margin plot tells the difference between the fractions of times a case is correctly classified and the fraction of times it is incorrectly classified. Sample 3 and Sample 20 have small margins, and it means these two samples have half of chances to be corrected classified and half of chance to be incorrectly classified.

**Figure 6:**
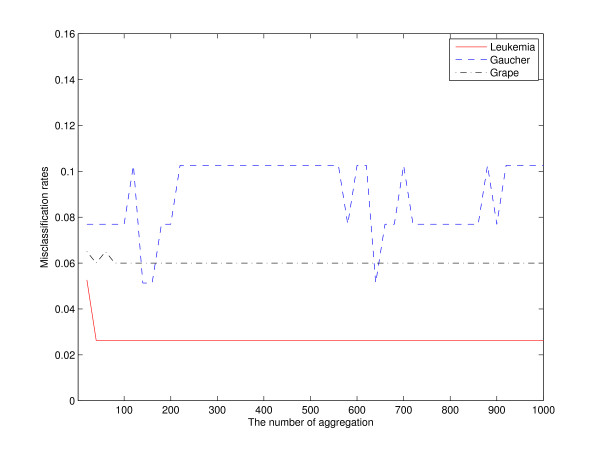
**The cross-validation errors in different number of aggregation**. In leukemia data, the misclassification rate keeps stable when the number of aggregating is more than 50. In grape data, the misclassification rate keeps stable when the number of aggregating is more than 100. In Gaucher data, the aggregating model is not stable.

Although the efficiency of aggregating depends on the stability of the prediction, aggregating does not definitely make the predictor stable, and it stabilizes to a certain extent. As shown in Figure 5, there is a small margin of sample 3 and sample 20 of the Gaucher proteomics data. The difference between the fractions of times a case is correctly classified and the fraction of times it is incorrectly classified is called the "margin" for that case [28]. Larger margins are desirable because a more stable classification of that sample is implied. As seen in Figure 5, some samples are always corrected predicted and also some samples (10 and 22) are always wrongly predicted. On the other hand, the small margins in sample 3 and 20 tell us that these two samples have almost half chances to be corrected classified, and half chances to be incorrectly classified. These two "instable samples" result in an aggregating classifier that is not stable. Figure 6 also supports such findings as the misclassification rates fluctuate greatly with different numbers of aggregation.

Another question about aggregating PCDA is how many times resampling is enough? Figure 6 gives the misclassification rate in training with increasing number of aggregation. The number of aggregation starts from 20 to 1000, and increases by 20 each time. We observe in Figure 6 that the aggregated misclassification rate will keep stable after 100 replicas in leukemia and grape data. For Gaucher data, 200 replicas also give a reasonable estimation. To our experience, 50-200 replicas are usually enough to get a stable value. Aggregating learner in this paper is obtained from cross-validation, which is resampling without replacement. The conventional bagging is obtained from bootstrapping, which is resampling with replacement. As stated by Buja and Stuetzel [30], there is an equivalence between bagging based on resampling with and without replacement. So, the conclusion obtained in this paper in our opinion also holds in bagging approaches.

Another concern is whether aggregating PCDA can apply to multi-classification problem. Because the discrimination in PCDA is performed by LDA, the properties of LDA for multi-classes also hold. Since the decision boundaries in LDA are constructed in a pair wise manner [1], the conclusions drawn in this paper in principle are also valid for a multi-class problem. However, many discriminative methods are often most accurate and efficient when dealing with two classes only, but usually at reduced accuracy and efficiency for multi-classification [31]. The effects of aggregating multi-classifier still need further careful studies.

In addition, an interpretable model is usually required as it is important to identify which genes, proteins and metabolites contribute most to classifiers. The PCDA model has been already combined with rank products [13,16,32] to find important variables. In aggregating PCDA, we can repeat the same strategy too. For example, we aggregate 100 PCDA learners together. As a single PCDA yields 10 discriminant vectors in a 10 fold cross-validation; 100 runs gives 1000 discriminant vectors in total. Then for all features the products of the 1000 ranks are calculated. After sorting, the features with the lowest rank products are the ones with the largest discriminative power.

## Conclusions

The use of cross-validation to study the performance of a classifier is an established method. If performed in a proper way cross-validation provides roughly unbiased estimates of the prediction measures. However, the different partitions in cross-validation can give rise to high variability of the model predictions. In this paper we show a way to overcome the variability by building one aggregated classifier from all the classifiers that were build in the repeating cross-validations.

Aggregating learners can have several important benefits. Aggregating over a collection of fitted values can help compensate for overfitting. That is, the majority voting tends to cancel out results shaped by idiosyncratic features of the data. One can then obtain more stable and more honest assessments of how good the fit really is.

Aggregating learners also have some limits. When the sample size is very small, aggregating learner may have a large bias. So it is important for us to visualize the data to see if aggregating will be helpful or not.

In conclusion, we recommend the use of aggregating learner in high dimensional data analysis, but a careful look on data structure and comparison with base learner result.

## Competing interests

The authors declare that they have no competing interests.

## Authors' contributions

All authors conceived the model and the structure of the paper. CJX performed the analysis and drafted the paper. All authors read and approved the final manuscript.
